# Recombinant IgG1 Fc-μTP-L309C Ameliorates Endogenous Rheumatoid Arthritis in the K/BxN Mouse Model by Decreasing Th1 and Th17 Cells in the Spleen, Lymph Nodes and Joint and Increasing T Regulatory Cells and IL-10 in the Joint

**DOI:** 10.3390/jcm14134509

**Published:** 2025-06-25

**Authors:** Bonnie J. B. Lewis, Selena Cen, Ruqayyah J. Almizraq, Beth Binnington, Rolf Spirig, Fabian Käsermann, Donald R. Branch

**Affiliations:** 1 Department of Laboratory Medicine and Pathobiology, University of Toronto, 30 Bond St., Toronto, ON M5B 1W8, Canada; bonnie.lewis2@griffithuni.edu.au; 2 Centre for Innovation, Canadian Blood Services, 30 Bond St., Toronto, ON M5B 1W8, Canada; selenacen1989@gmail.com (S.C.); beth.binnington@blood.ca (B.B.); 3 Bluerock Therapeutics, 101 College St., Toronto, ON M5G 0A3, Canada; ralmizraq@bluerocktx.com; 4 CSL Behring Biologics Research Center, 200031 Bern, Switzerland; rolf.spirig@cslbehring.com (R.S.); fabian.kaesermann@cslbehring.com (F.K.); 5 Department of Medicine, University of Toronto, 30 Bond St., Toronto, ON M5B 1W8, Canada

**Keywords:** arthritis, K/BxN mice, recombinant Fc-hexamer, human IgG1 rFc-µTP-L309C, Th17 cells, T-regulatory cells, interleukin-1

## Abstract

**Background/Objectives:** Recombinant Fc proteins have been produced that have a protective effect in mouse models of arthritis, such as the K/BxN rheumatoid arthritis model. We have previously shown that a recombinant human IgG1 Fc with a point mutation at position 309, replacing a leucine with a cysteine, fused to the human IgM tailpiece to form a human IgG1 Fc hexamer, rFc-µTP-L309C, effectively prevents neutrophil infiltration into the joints and ameliorates arthritis in the K/BxN serum transfer model and in the endogenous chronic arthritis K/BxN model. We have now investigated the effect of rFc-µTP-L309C on T-cells in the K/BxN chronic arthritis mouse model. **Methods:** PBMCs were isolated from the spleen, lymph nodes and joint synovial fluid from K/BxN mice having severe chronic arthritis that had been treated with 200 mg/kg rFc-µTP-L309C or human serum albumin (HSA). Flow cytometry was used to isolate the activated CD4^+^CD44^+^ T-cells and T-regulatory cells (Tregs). Intracellular staining was used to identify Th1 and Th17 T-cell subsets, and CD4^+^CD25^+^FoxP3^+^ Tregs. ELISA was used to measure levels of IL-10 and TGF-β in synovial fluid. **Results:** We find that amelioration of the arthritis occurs after treatment with rFc-µTP-L309C and results in a decrease in Th1 cells’ production of IFNγ and Th17 cells’ production of IL-17. Amelioration also results in decreased production of GM-CSF. Moreover, amelioration results in increased Tregs and IL-10 production in the synovial fluid. **Conclusions:** rFc-µTP-L309C reduces the inflammatory T-cells and increases the regulatory anti-inflammatory T-cells in the chronic arthritis K/BxN mouse model. This effect explains, in part, the ability of rFc-µTP-L309C to ameliorate the arthritis and reduce damage on the articular cartilage of K/BxN mice.

## 1. Introduction

Serum transfer experiments containing arthritogenic antibodies from the K/BxN mouse model of rheumatoid arthritis has been used extensively to examine the mechanism of amelioration of the arthritis by intravenous immunoglobulin (IVIG) [1,2,3,4,5,6,7,8,9]. These publications have used a serum-transfer approach by obtaining arthritogenic serum from severely affected mice and then transferring those sera into normal, non-arthritic mice [1,2,3,4,5,6,7,8,9]. We have previously reported on using the K/BxN mouse model to examine the effects of IVIG and recombinant IgG1 rFc-μTP-L309C hexamer on amelioration of the endogenously produced arthritis in this model [6]. We found that either IVIG or rFc-μTP-L309C were efficacious to significantly ameliorate the arthritis in these mice with rFc-μTP-L309C 10-fold more efficacious than IVIG [6]. We further showed that the mechanism of reversal of arthritis was, in part, due to rFc-μTP-L309C able to inhibit neutrophil infiltration into the joints as well as inhibition of IL-1β production [10].

Our earlier work looking at the effect of rFc-μTP-L309C treatment on neutrophils [10] provided a partial explanation as to the mechanism of action of rFc-μTP-L309C to ameliorate the arthritis in the K/BxN mouse model. However, arthritis is a multifactorial disease, and we wished to understand what role, if any, the T-cell compartment may play in the effect of rFc-μTP-L309C on ameliorating the arthritis. Thus, in this brief correspondence, we have further investigated the possible mechanism of rFc-μTP-L309C to ameliorate endogenous arthritis by examining the effects of this molecule on T-cells. We find that treatment with rFc-μTP-L309C causes a decrease in the population of the T-helper 1 (Th1) and Th17 phenotypes with decreases in production of interleukin-17 (IL-17) and interferon-gamma (IFN-γ), in the spleen, lymph nodes and joints. Granulocyte-macrohage-colony stimulating factor (GM-CSF) was, in fact, increased in the spleen but was significantly decreased in the other tissues, including the joints. Concomitant increases in FoxP3^+^ T-regulatory (Tregs) cells and IL-10 were also found in the joint synovial fluid. Transforming growth factor-β (TGF-β), although increased, the increase was not found to be significant. The decrease in the inflammatory Th1 and Th17 cell populations and an increase in anti-inflammatory Tregs, IL-10 and, marginally, TGF-β explains, in part, the mechanism of rFc-μTP-L309C amelioration of the arthritis in these animals.

## 2. Materials and Methods

### 2.1. K/BxN Mice

K/BxN mice were described previously as a mouse model of chronic rheumatoid arthritis [11]. The model is generated by crossing a T-cell transgenic line (KRN) with the nonobese diabetic NOD mouse strain [12]. KRN T-cell receptor (TCR) transgenic mice on a C57BL/6 background were obtained from the Jackson Laboratory, a kind gift from C. Benoist, Harvard University. NOD/Lt mice were purchased from The Jackson Laboratory. Arthritic mice were obtained by crossing KRN mice (F, 6 weeks old) with NOD/Lt (M, 6 weeks old) mice to produce K/BxN mice expressing both the TCR transgene KRN and the major histocompatibility complex (MHC) class II molecule I-Ag7. Mice were kept under a natural light-dark cycle, maintained at 22 ± 4 °C, and fed with standard diet and water ad libitum. The resulting K/BxN mice, were kept for 21 days post weaning when arthritis symptoms first develop and allowed to attain an arthritis clinical score of 9–12 prior to starting treatments [6,10,12]. All experiments were performed following our institutions’ guidelines on the care and use of laboratory animals and only after approval of the protocol by the animal care committee of the University Health Network (UHN); animal use protocol AUP 1788, and after review by the St. Michael’s Hospital Unity Health Toronto Animal Care Committee (ACC); approved protocol ACC 138.

### 2.2. Reagents

Recombinant Fc-µTP-L309C was provided by CSL Behring AG, Switzerland. Human serum albumin (HSA) was obtained from Canadian Blood Services (CBS),Ottawa, ON, Canada. Antibodies used for flow cytometry were: anti-cluster of differentiation (CD)44-PE-Cy7 (clone IM7), anti-GM-CSF-FITC (clone MPI-22E9), anti-CD25-APC (clone PC61) and anti-IFN-γ-Pacific Blue (clone XMG1.2) from Biolegend, San Diego, CA, USA, anti-CD4-APC-Alexa 750 (clone RM4-5), anti-IL-17-APC (clone eBio-17B7), and anti-FOXP3-FITC (clone FJK-16s) from Thermofisher Scientific, Waltham, MA, USA). Cascade Yellow viability stain was from Thermofisher Scientific. For Fc blocking, anti-CD16/32 (clone 2.4G2) was purchased from BD Pharmingen, San Jose, CA, USA. PMA and ionomycin were purchased from Sigma Aldrich, St. Louis, MI, USA. GolgiStop was from Thermofisher Scientific. Enzime-linked immunosorbet assay (ELISA) kits for IL-10 and transforming growth factor (TGF)-β determination were from R&D Systems (Minneapolis, MN, USA).

### 2.3. Arthritis Scoring and Treatment

Arthritis scoring has been previously described [6,12]. The paws of the mice were monitored daily using digital calipers over the course of each experiment [6,10]. The development of arthritis was assessed daily, and the severity of arthritis was scored for each paw on a 3-point scale, in which 0 = normal appearance, 1 = localized edema/erythema over one surface of the paw, 2 = edema/erythema involving more than one surface of the paw, 3 = marked edema/erythema involving the whole paw. The scores of all four paws were added for a composite score, with a maximum score of 12 per mouse [6,10,12]. K/BxN mice with high clinical scores of 9 or greater were treated by subcutaneous injection (s.c) of 200 mg/kg rFc-µTP-L309C on days 1, 3, 5, 7, 9, and 11. HSA was used as a protein control.

### 2.4. Organs Examined

The spleens, popliteal lymph nodes, and joints from K/BxN mice that received 6 s.c. injections of 200 mg/kg of rFc-µTP-L309C were isolated and placed in Roswell Park Memorial Institute (RPMI) 1640 + 5% FCS on ice. Mice treated with human serum albumin (HSA, purchased from CBS) were used as a control. The spleens and popliteal lymph nodes were pushed through a fine mesh strainer (70-µm cut-off) to form a single cell suspension. Red blood cells (RBCs) were lysed from the spleens. The cells were washed and resuspended in phosphage buffered saline (PBS) + 2% fetal bovine serum (FBS) for cell counts. Joint washes were performed as previously described [13]. Malleoli and surrounding soft tissue (excluding fat) were removed from both rear limbs and placed in RPMI 1640 + 5% FCS on ice for 60 min. The medium was then removed, centrifuged, and the supernatant (joint wash) stored at −20 °C until subsequent analysis. The washed malleoli and cell pellets were combined for each individual mouse and digested for 30 min at 37 °C with 1 mg/mL collagenase (CLS-1, 250 U/mg; Worthington Biochemical, Lakewood, NJ, USA) and 0.1 mg/mL DNase I (1U/µL; Thermo Fisher). The digests were strained (70-µm cut-off), washed, and resuspended in PBS + 2% FCS for cell counts. Tregs were enriched using the EasySep mouse Treg cell enrichment kit (Stemcell Technologies, Vancouver, BC, USA). Single-cell suspensions of spleens, popliteal lymph nodes and joint digests were resuspended in PBS containing 2% FCS. Cells were incubated with anti-CD16/32) as Fc-gamma receptor (FcγR) blocking agent and stained for CD4, CD25 and/or CD44. Intracellular staining was used for GM-CSF, IL-17, IFN-γ, and/or FOXP3. For intracellular staining, cells were stimulated with phorbol myristate acetate (PMA) and ionomycin GolgiStop was used to enhance the detection of the intracellular stains. Cells were permeabilized with a FOXP3 permeabilization kit (Thermofisher Scientific). Cells were fixed with 4% paraformaldehyde (PFA) and staining was analyzed by flow cytometry on a BD LSRFortessa (BD Biosciences, San Jose, CA, USA), and the data was analyzed by using FlowJo software (FlowJo™ v10) (BD Biosciences, Ashland, OR, USA). ELISA kits for measurement of IL-10 and TGF-β were used according to manufacturer’s protocols. Mice treated with HSA at a similar dose as for rFc-µTP-L309C were used as a control.

### 2.5. Statistical Analysis

Statistical tests were performed using GraphPad Prism 8 for Windows software. To compare clinical scores across treatment groups the Kruskal-Wallis test was performed with Dunn’s post test to compare pairs of data. For Dunn’s multiple comparison test, an adjusted *p*-value < 0.05 was considered statistically significant. Differences in cell populations identified by flow cytometry (treated vs. control) were identified using the Mann-Whitney test, with *p* < 0.05 considered significant. Data in all graphs is shown as mean ± standard deviation (SD).

## 3. Results

*3.1. rFc-µTP-L309C Ameliorates Endogenous Arthritis in K/BxN Mice*

**Figure 1:**
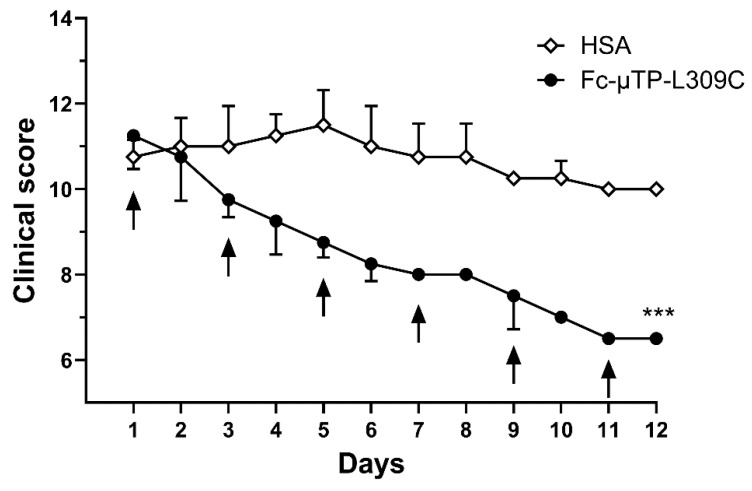
The clinical scores are shown for mice treated with multiple doses of 200 mg/kg of Fc-µTP-L309C, using HSA-treated mice as a control. Arrows indicate treatments given on days 0, 1, 3, 5, 7, 9, and 11. Error bars indicate standard deviation of mean clinical scores; (*n* = 4 for each treatment group) and are shown in one direction for clarity. *** *p* < 0.001 rFc-µTP-L309C vs. HSA control using Kruskal–Wallis with Dunn test.

Figure 1 shows the efficacy of rFc-µTP-L309C to ameliorate spontaneously acquired, a rthritis in K/BxN mice. Severe arthritis was allowed to develop to a clinical score of ~10 (day 1), then 200 mg/kg rFc-µTP-L309C or HSA as a control was administered for six treatments given on days 1, 3, 5, 7, 9, and 11. Final evaluation was on day 12, at which time control, HSA-treated mice, reached an average clinical score of 10 while the clinical score of the rFc-µTP-L309C-treated mice had dropped to 6.5 (*p* < 0.0001).

### 3.2. Th1 and Th17 T-Cells Are Decreased in the Spleen, Lymph Nodes and Joints of K/BxN Mice After rFc-µTP-L309

K/BxN mice with high clinical arthritis scores of 9–12 were given 6 s.c. injections of 200 mg/kg of rFc-µTP-L309C or HSA on days 1, 3, 5, 7, 9, and 11. The spleen, popliteal lymph nodes and ankle joint fluid were collected (after dissection) on day 12 for T-cell subset analysis by flow cytometry. Knee joint synovial fluid was also collected for an ELISA to measure IL-10 and TGFβ levels.

**Figure 2:**
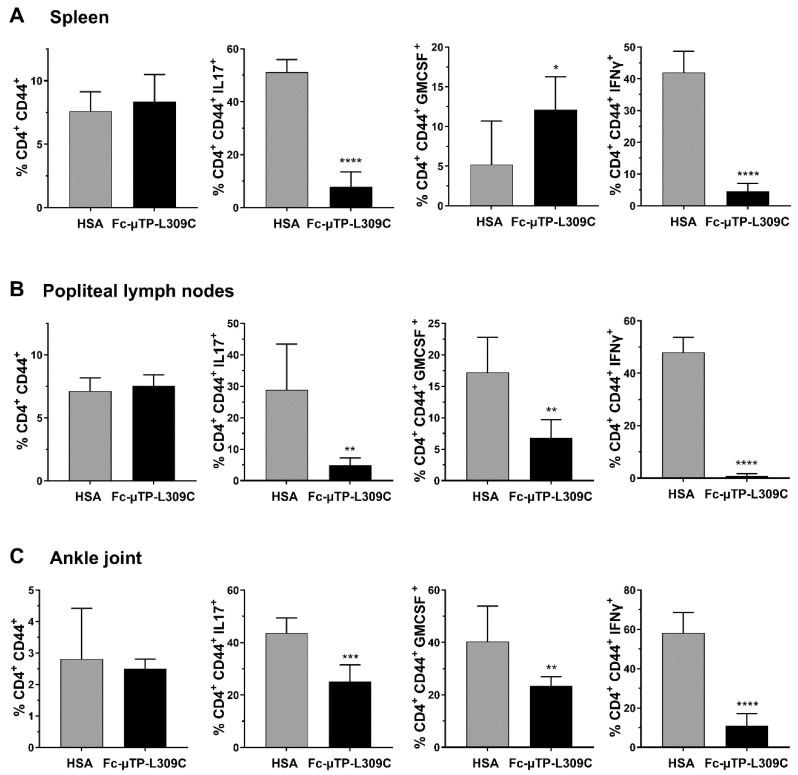
rFc-µTP-L309C effects on cytokine-expressing T-cells in the spleen, popliteal lymph nodes and ankle joint. The indicated tissues were collected on day 12 from K/BxN mice treated with 6 s.c. injections of 200 mg/kg HSA (gray bars) or rFc-µTP-L309C (black bars). Percentage (%) of CD4^+^ CD44^+^ T-cells expressing intracellular GM-CSF, IL-17 or IFN-γ in the spleen (**A**), popliteal lymph nodes (**B**) or ankle joint fluid (**C**) were measured. Error bars represent standard deviation of mean values (*n* = 6–8 per treatment group. * *p* < 0.05, ** *p* < 0.01, *** *p* < 0.001, **** *p* < 0.0001 using Mann-Whitney test.

Similar numbers of activated T-cells (CD4^+^CD44^+^) were seen in the spleen (Figure 2A), popliteal lymph nodes (Figure 2B) and ankle joints (Figure 2C). Using intracellular staining we show that IL-17 expressing cells (marker for Th17 T-cells) were significantly decreased in the spleen, popliteal lymph nodes and ankle joints (Figure 2A–C). GM-CSF expressing cells were significantly increased in the spleen (Figure 2A), and decreased in popliteal lymph nodes and ankle joints (Figure 2B,C). IFN-γ expressing cells were substantially decreased (*p* < 0.0001) in all three tissues examined (Figure 2A–C) GM-CSF and IFN-γ aremarkers for Th1 T-cells).

### 3.3. Tregs and IL-10 Are Increased in the Spleen, Lymph Nodes

**Figure 3:**
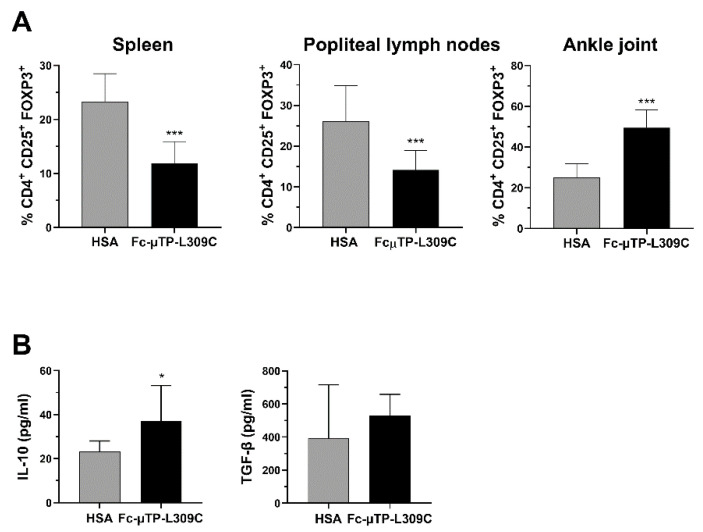
rFc-µTP-L309C effects on Tregs, IL-10 and TGF-β. Spleen, popliteal lymph nodes and ankle joint fluid were collected on day 12 from K/BxN mice treated with 6 s.c. injections of 200 mg/kg HSA or rFc-µTP-L309C. (**A**) Percentages of FoxP3^+^ Tregs in spleen, popliteal lymph nodes and ankle joint, compared to total CD4^+^ cells. Error bars represent the standard deviation of mean values (*n* = 9) *** *p* < 0.001, compared with HSA using a Mann-Whitney test. (**B**) ELISA measurement of IL-10 and TGFβ in synovial fluid from knee jointfrom mice treated as in (**A**). Shown are the average IL-10 and TGF-β concentrations measured in pg/mL; error bars indicate the standard deviation of mean values (*n* = 3 per treatment group). * *p* ˂ 0.05, compared with HSA using a Mann-Whitney test.

We found that the number of CD4^+^CD25^+^FOXP3^+^ Tregs were significantly decreased in the spleen and popliteal lymph nodes but, in contrast, significantly increased in the joint (Figure 3A). In line with these results, we found that the level of IL-10 in the synovial fluid of these mice was also higher after repeated administration of rFc-µTP-L309C compared with HSA (Figure 3B, left). Although TGF-β showed an increase in rFc-µTP-L309C-treated mice (mean ~500 pg/mL compared to ~400 pg/mL for has-treated), this moderate trend increase was not significant (Figure 3B, right).

## 4. Discussion

We have previously shown that the arthritis produced endogenously by K/BxN mice can be ameliorated by either IVIG or rFc-µTP-L309C, a recombinant human IgG1 Fc-hexamer [6,10]. Although rFc-µTP-L309C was shown to inhibit neutrophil infiltration into the joint as well as IL-1β, and this effect was undoubtedly responsible, at least in part, for the amelioration of the severe arthritis in these mice, it didn’t provide a complete understanding of possible mechanisms for the therapeutic response of the molecule. In the study herein, we have further examined in the K/BxN arthritis model, the effect of rFc-µTP-L309C on the expression of T-cell subsets, including Th1, Th17 and T-regulatory cells (Tregs) in the spleens, lymph nodes and joints, as well as production of anti-inflammatory molecules, IL-10 and TGF-β, in the joint synovial fluid. We have determined that treatment with rFc-µTP-L309C results in a significant decrease in Th1 and Th17 cells in the joints and a concomitant increase in Tregs and production of IL-10. Interestingly, the increase of Tregs is seen only in the joints; indeed, in contrast, Tregs are found to be decreased in the spleen and lymph nodes. Perhaps, this apparent dichotomy indicates that Tregs, as previously suggested [14], in response to rFc-µTP-L309C, are migrating from the spleen and lymph nodes into the joints to protect the joint? The observed increase in IL-10 is presumably because of the increase in Tregs (Figure 3B).

Tregs play a major role in mediating the humoral response through the suppression of autoantibody production by B cells and production of anti-inflammatory mediators such as IL-10 and TGFβ [14,15,16,17]. Nguyen et al. [14] reported a role for Tregs in Ab-induced arthritis at several levels. First, they showed that a Treg deficiency in K/BxN mice led to more accelerated aggressive arthritis with significantly earlier auto-Ab production. They also showed that Tregs accumulated in the inflamed joints of K/BxN serum-transferred C57BL/6 mice, which suggested that Tregs actively migrate to the site of Ab-induced inflammation and control the local inflammatory process. The main ways in which these Tregs control the local inflammatory response is through the secretion of IL-10 and TGF-β [14,16,17]. IL-10 has inhibitory effects on cells of the innate immune system through suppressing pro-inflammatory cytokine and chemokine production by activated monocytes/macrophages and neutrophils [14,16,17], whereas TGF-β is more known for its effects on B cells such as its ability to suppress B cell survival, proliferation, differentiation into plasmablasts, and antibody secretion. Although there is some evidence to suggest that IL-10 can indirectly affect Ab secretion by B cells by modulating B cell metabolite [16], more studies are needed to determine whether this is true. In our study, rFc-µTP-L309C was able to increase the Tregs in the joints as well as IL-10. Some studies have shown that TGF-β is important for the amelioration of arthritis in the joints [18,19].

Although TGFβ was increased in the joints of the mice, this increase was not significant. Nonetheless, taken together our results support previous published work that Tregs, IL-10 and TGF-β play a significant role in the arthritis [16,17,18,19] and our findings, along with decreased numbers of inflammatory Th1 and Th17 cells, can explain, at least in part, the amelioration of the endogenous arthritis by rFc-µTP-L309C in our K/BxN mouse model. How human rFc-hexamer can reduce the impact of the T-cell phenotype is uncertain; however, it may be by engaging FcγR myeloid cells to secret factors, i.e., cytokines, which impact the T-cell phenotype?

It is possible that the effects we see on the T-cell compartment are mediated by Fcγ receptors (FcγRs) on monocyte-macrophages or dendritic cells. rFc-µTP-L309C has been shown to bind to Fcγ receptors with high affinity and avidity [4]. Thus, FcγRs interactions with rFc-µTP-L309C may be responsible for some of the outcomes we have described. rFc-µTP-L309C can bind to all activating FcγRs, including FcγRI, FcγRIIa and FcγRIIIa. It also can bind to the inhibitory Fcγ receptor, FcγRIIb. Perhaps the dynamics of the crosstalk between activating and inhibitory FcγRs might determine the balance between tolerance (generation of Tregs and anti-inflammatory cytokines) and autoimmunity (generation of Th17 cells) [20].

There are some limitations to our work. The use of a single animal model (K/BxN) is a limitation in terms of generalizability to other RA models or humans. Also, we did not look at the mechanism of the effects on the T-cell populations. The effects could be mediated by rFc-µTP-L309C interactions with Fc receptors on macrophages or dendritic cells. TGF-β findings are not definitive and should be looked at more carefully with more animals. These aspects could be addressed in future experiments.

In conclusion, rFc-µTP-L309C ameliorates chronic arthritis in the K/BxN mouse model by multiple mechanisms, including affects on neutrophil infiltration into the joints and inhibition of IL-1β as previously described [7]. In the work herein, we describe another mechanism that rFc-µTP-L309C affects, the T-cell compartment. rFc-µTP-L309C decreases inflammatory cytokines by inhibiting Th1 and Th17 cells and increases T regulatory cells and anti-inflammatory IL-10.

## Data Availability

The datasets used and/or analyzed during the current study are available form the corresponding author. All data generated or analyzed during this study are included in this publication.
